# Acute Effects of a Single Football Training or Match on Passive Hip Rotation Range of Motion in Semi-Professional Football Players: A Pilot Study

**DOI:** 10.3390/medicina56050228

**Published:** 2020-05-10

**Authors:** Blanca De-la-Cruz-Torres, Vanesa Abuín-Porras, Maria Blanco-Morales, Mónica de-la-Cueva-Reguera, César Calvo-Lobo, Daniel López-López, Carlos Romero-Morales

**Affiliations:** 1 Department of Physiotherapy, University of Seville, Avicena Street, 41009 Seville, Spain; 2 Faculty of Sport Sciences, Universidad Europea, Villaviciosa de Odón, 28670 Madrid, Spain; vanesa.abuin@universidadeuropea.es (V.A.-P.); maria.blanco@universidadeuropea.es (M.B.-M.); monica.delacueva@universidadeuropea.es (M.d.-l.-C.-R.); carlos.romero@universidadeuropea.es (C.R.-M.); 3 Facultad de Enfermería, Fisioterapia y Podología, Universidad Complutense de Madrid, 28040 Madrid, Spain; cescalvo@ucm.es; 4 Research, Health and Podiatry Group, Department of Health Sciences, Faculty of Nursing and Podiatry, Universidade da Coruña, 15403 Ferrol, Spain; daniel.lopez.lopez@udc.es

**Keywords:** hip, soccer, load, risk factor, injury prevention

## Abstract

*Background and Objectives*: The repetitive loading forces generated during football activities may induce alterations in the hip rotation range of motion (ROM) in players. The objective of this study was to evaluate the acute effects of a training and a match on bilateral passive hip rotation ROM in both lower limbs in soccer. *Material and Methods*: Twenty-eight male players were divided into two groups: 14 players (28 limbs) with normal bilateral hip rotation ROM (NH group) and 14 players (28 limbs) with restricted bilateral hip ROM (RH group). Passive bilateral hip rotation ROM was measured, by goniometer, before and after training or a match. Internal-rotation ROM (ROM_IR_), external-rotation ROM (ROM_ER_), total ROM (ROM_TOT_) and relative internal rotation (ROM_REL_) were calculated. *Results*: The NH group did not show substantial changes in hip ROM after a training nor a match. After a training session, only the RH group exhibited a substantial increase in ROM_IR_, ROM_ER_ and ROM_TOT_. After a match, only the RH group exhibited a substantial increase in ROM_ER_ and ROM_TOT_ and exhibited a substantial decrease in ROM_REL_. Comparing both groups, there were significant differences within ROM changes for ROM_ER_ and ROM_TOT_ after training and for ROM_ER_ and ROM_REL_ after a match. *Conclusions*: Despite the small sample size of the present study, the findings indicate that a single football activity leads to significant changes in hip rotation ROM in players with restricted bilateral hip external-rotation ROM. However, these changes did not reach reference cut-off scores.

## 1. Introduction

Football is the world’s most popular sport and repeated high intensity movements (including acceleration, deceleration, jumping, landing tasks and rapid changes of direction) are required during football activities [1,2,3]. These movements may cause an overload in the joints, generating a biomechanical imbalance that could affect their normal range of motion (ROM), both in training sessions and games. Any biomechanical alterations, especially in the lower extremity, may disturb proper motor development and cause pain when playing sports [4,5]. In this regard, Sahrmann [6] identifies motion as a cause of alterations and tissue damage, especially when the movement deviates from the optimal kinesiological pattern.

Because of different lower extremity demands among sports, passive hip ROM may differ by sport [7]. In football, Mosler et al. [8] proposed normal values for hip ROM in professional football players (internal rotation in prone 38 ± 8° and external rotation in supine 32 ± 8°); Manning and Hudson [9] suggested that a specific pattern of hip ROM, measured in supine, was found in professional footballers (internal rotation in supine 25° and external rotation in supine 44°), which appeared to be different from controls (internal rotation 43° and external rotation 43°); López-Valenciano et al. [10] found non-clinically relevant bilateral differences (>6°) between the dominant (internal rotation 47.1 ± 8.0° and external rotation 49.9 ± 9.8°) and non-dominant (internal rotation 45.3 ± 7.9° and external rotation 50.7 ± 9.8°) hip joints’ ROM average values in football players, measured in prone. Some authors [8,9,11] suggested that group average ROM may distort the number of players reporting restricted ROM due to the fact that there is a large degree of inter-player variability in ROMs. Experts support the notion that there are football-specific adaptations in hip rotation ROM, but there are limited studies on the effect of football activities on hip ROM [9,10]. The restricted scores have been considered as primary risk factors for some of the most common injuries in football. Tak et al. [12] obtained that a decreased hip ROM in professional soccer players was associated with more hip- and groin-related symptoms. King et al. [13] compared the hip biomechanics associated with femoro-acetabular impingement syndrome and rotation ROM between both limbs of sub-elite football players with unilateral hip-related groin pain. They concluded that these players had less hip ROM_ER_ and no difference in rotation ROM_IR_. Verrall et al. [14] described a reduction in hip rotation ROM in players with chronic groin injury diagnosed as pubic bone stress injury.

Thus, it is necessary to clarify whether the repetitive loading forces generated during a single football training and/or match induce alterations in the normal and restricted hip rotation ROM in football players. This finding may help sports scientists and therapists to establish preventive programs, which include hip rotation measurements or treatments, that may be included as part of a football player’s training and/or match to potentially reduce the risk of player injury.

Therefore, since rotational hip profiles have not been evaluated after a single football training session or competitive match in semi-professional male football players, the purpose of this study was to evaluate the acute effects of a training and a competitive football match on bilateral passive hip rotation ROM in the dominant and non-dominant hip of semi-professional football players.

## 2. Materials and Methods

### 2.1. Participants

The power calculation (using the G* Power software- version 21) was based on the total rotation variable of an initial samples (*n* = 8) with two groups (mean ± SD), 4 subjects with restricted bilateral hip ROM (67 ± 3.75) and 4 with normal bilateral hip rotation ROM (74 ± 4.79). Indeed, 1-tailed hypothesis and effect size of 1.52 and an α error probability of 0.05, a power (1 − β error probability) of 80% and an allocation ratio (*N*2/*N*1) of 1 were applied for the sample size calculation. Therefore, a total sample size of 14 subjects was calculated.

Twenty-eight semi-professional male football players from Spanish honor division clubs volunteered for the study (Table 1). In each participant, the hip rotation ROM scores were categorized as normal or restricted according to the reference values previously reported to consider an athlete as being more prone to suffer an injury [15,16]. ROM values were reported as restricted according to the following cut-off scores: <35° for the ROM_ER_ [17]. They were divided into two groups: one made up of 14 players (28 limbs) with normal bilateral hip rotation ROM (NH group) and the other made up of 14 players (28 limbs) with restricted bilateral hip ROM_RE_ (RH group).

An initial examination by the sport doctor focused on orthopedic and other conditions that might preclude study, and all the participants were found to be in good health (for example, structural abnormalities were refused). Inclusion criterion for the NH group included: (i) a minimum of 10-years football experience; (ii) semi-professional level; and (iii) no low back or lower extremity injury in the previous 3 months. For the RH group, the inclusion criteria were: (i) a minimum of 10-years football experience; (ii) semi-professional level; (iii) restricted bilateral hip ROM_RE_, caused by an imbalance between the internal and external rotator muscles and characterized by a shortening of the internal rotator muscles [6] (this imbalance is due to a lack of stretching of the hip muscles and lumbopelvic exercise that keeps the hip joint balanced [6]) and (iv) functional alteration of the hip. Exclusion criterion included: (i) having musculoskeletal injuries during the previous three months; (ii) current active disease processes; (iii) structural alteration of the hip; or (iv) previous surgery.

### 2.2. Ethics

The study was approved by the ethics committee of the University Hospital Virgen Macarena-Virgen del Rocio (19 June 2019) in accordance with the Declaration of Helsinki. Before enrolling, the subjects were fully informed about the protocol and a written informed consent was obtained.

### 2.3. Testing Procedures

All passive bilateral hip ROM measurements were carried out by an experienced physiotherapist with more than 10 years of experience. The examiner was blind to the group assignment and the mean of the two values of each variable was used for the corresponding analysis. All variables were obtained using a universal goniometer, as previously described [8]. All players were positioned prone with the hip in 0° of extension and 30° of abduction. This neutral hip positioning minimized muscular involvement. The knee was flexed to 90°. The leg was passively rotated internally and externally until initial sacral tilt as determined by the physiotherapist’s palpation. At this point, the transverse angle formed by true vertical and the tibial diaphysis were considered ROM_ER_ and ROM_IR_, respectively. ROM_TOT_ was considered as the sum of ROM_ER_ and ROM_IR_. Total hip ROM is the factor most consistently related to groin pain in athletes [18]. The subtracted ROM_ER_ from ROM_IR_ was used to calculate ROM_REL_. This relative ROM_IR_ variable retained the original degree unit and has been used in the literature to represent the amount of bias toward ROM_IR_ [19]. All tests were performed before and after a training session or official match. The initial measurements were evaluated before the warm-up of the training or match and the final scores were measured just after training or the match (the examiner took no more than 5 min).

### 2.4. Statistical Analysis

The normality of the data distribution was verified by the Shapiro–Wilk test. Descriptive statistics (mean ± SD) for the different variables were calculated. Paired *t* tests confirmed no appreciable differences in bilateral ROM_IR_ or ROM_ER_.

Repeated-measures analysis of variance (ANOVA) with 2 factors, 2 (group) × 2 (time: pre-training and post-training), were performed for ROM_IR,_ ROM_ER,_ ROM_TOT_ and ROM_REL_. To interpret the effect size (ES) results, Cohen suggested that *d* = 0.02 was considered “small”, *d* = 0.5 was considered “medium” and a *d* = 0.8 was considered a “large” effect size [20].

The intraclass correlation coefficient (ICC) and the standard error of measurements (SEM = SD/√n) were used to determine the intra-observer reliability of the measurements.

## 3. Results

All football players were healthy and competing at the time of the trial, training and playing a total of 12 h per week of football and having a previous average football experience of 19.42 ± 4.69 years.

There were no significant baseline differences between both groups in any participant characteristics variables (Table 1). There were also no differences between the duration of training and the match (warm up + training: 108.75 ± 6.66 min; and warm up + match: 112.00 ± 10.69 min, *p* = 0.26).

### 3.1. Internal Rotation

Variations in hip ROM_IR_ of each group after the training or match are presented in Table 2 and Table 3. The NH group did not show substantial changes after training nor the match. The RH group showed significant differences after training (*p* = 0.01; *d* = 0.6) but not after the match.

No differences were observed after training or the match in ROM_IR_ changes between groups (Table 4). The ICC and SEM were 0.94 and 2.89, respectively, for the hip ROM_IR_ measurements, indicating high reliability.

### 3.2. External Rotation

Variations in hip ROM_ER_ of each group after the training or match are presented in Table 2 and Table 3. The NH group did not show substantial changes after the training nor match. The RH group showed significant differences after the training (*p* = 0.001; *d* = 2.0) and match (*p* = 0.003; *d* = 1.0).

Differences were observed after the training (*p* = 0.001; *d* = 0.3) and match (*p* = 0.01; *d* = 1.1) in ROM_ER_ changes among the groups (Table 4). The ICC and SEM were 0.85 and 2.18, respectively, for the hip ROM_ER_ measurements, indicating good reliability.

### 3.3. Total Rotation

Variations in hip ROM_TOT_ of each group after the training or match are presented in Table 2 and Table 3. The NH group did not show substantial changes after the training nor match. The RH group showed significant differences after the training (*p* = 0.001; *d* = 1.4) and match (*p* = 0.03; *d* = 0.2).

Differences were observed after training (*p* = 0.006; *d* = 0.2) in ROM_TOT_ changes among the groups. However, no differences were observed after the match between groups (Table 4). The ICC and SEM were 0.85 and 4.21, respectively, for the hip ROM_TR_ measurements, indicating good reliability.

### 3.4. Relative Internal Rotation

Variations in hip ROM_REL_ of each group after the training or match are presented in Table 2 and Table 3. The NH group did not show substantial changes after the training nor match. The RH group showed significant differences after the match (*p* = 0.05, *d* = 0.7) but not after training.

No differences were observed after training in ROM_REL_ changes between both groups. However, differences were observed after the match between groups (*p* = 0.03; *d* = 0.0) (Table 4). The ICC and SEM were 0.94 and 4.15, respectively, for the hip ROM_REL_ measurements, indicating high reliability.

## 4. Discussion

The main finding of this study was that football activities mainly affected the hip rotation ROMs of players with restricted hip ROM_ER_. On the one hand, a football training session induces an immediate elevation in ROM_IR_ (moderate ES), ROM_ER_ (large ES) and ROM_TOT_ (large ES) in the RH group. However, the data only obtained normal values of total rotation due to the increase in internal rotation after training. On the other hand, a football match induces an immediate elevation in ROM_ER_ (large ES) and ROM_TOT_ (small ES) as well as an immediate decrease in ROM_REL_ (moderate ES) in the RH group. In this case, the data did not obtain normal values of ROM_ER_ and ROM_TOT_ because there were no changes in ROM_IR_ (Table 2 and Table 3). Therefore, the functional alteration of the hip in the players with restricted hip ROM_EM_ was maintained during football activities. These data demonstrate that players with restricted bilateral hip ROM_RE_ maintained their pathological values despite the fact that football activities improve hip rotation ROMs due to tissue warming. Therefore, players maintain an alteration of movement in the hip that may be more predisposing to injuries during training or a match. In fact, there were significant mean differences of changes in favor of the NH group, both after training and the match (Table 4). We suggest that football activities caused changes in the hip rotation ROM in players with restricted bilateral hip ROM_RE_, and that these changes were enough to reach normal ROM values. Altered movement patterns exposed repetitive and high loading forces generated during sports have been suggested to contribute to a variety of musculoskeletal pain conditions [12,13,14]. Therefore, these players were at risk of injury, mainly after a match. According to Sahrmann [6], authors suggest that the cumulative effect of repetitive actions (especially when their performance deviates from the optimal kinesiological pattern) causes tissue damage. Furthermore, authors hypothesize that musculoskeletal disorders of a mechanical origin may be relieved and prevented by correcting motion patterns and tissue adaptations. Therefore, these findings highlight the relevance of measuring the hip rotation before football trainings and matches in order to keep hip health.

In recent years, increasing evidence is now demonstrating that it is necessary to identify biomechanical deficiencies in the joints before sport activities. Accordingly, studies have now been published on the role of the hip ROMs in different sports. They tried to correlate these parameters with the development of subsequent injuries. Li et al. [21] showed higher rates of hip, groin and hamstring injuries in players with decreased hip internal rotation. King et al. [11] identified that football players with unilateral hip-related groin pain had less hip ROM_ER_ and no difference in rotation ROM_IR_. Tak et al. [10] suggested that a decreased hip ROM in professional soccer players was associated with more hip- and groin-related symptoms. Nevertheless, limitations in the range of hip movement may have negative effects on distal joints. Verrall et al. [12] described a reduction in hip ROM was evident in athletes with chronic groin injury diagnosed as pubic bone stress injury. Scher et al. [22] correlated altered hip ROM with shoulder injuries in professional pitchers and Saito et al. [23] found that a decreased hip flexion and internal rotation (IR) were risk factors for elbow pain. We hypothesized that the hips are a key component to performance and injury risk.

Although the relationship between hip ROM and injuries is gaining interest, the understanding of the evolution of hip ROM after trainings, after matches or over the course of the football season remains limited. Accordingly, our results showed that a football training session or match induced an acute increase of the ROM_ER_, yet ROM_ER_ did not reach the normal range in those with baseline abnormal ROM_ER_. Similarly, previous work showed that football players with lower preseason ROM in the hip flexors and knee flexors had a higher risk for muscle injuries to these muscle groups [24]. Paul et al. [25] obtained that a threshold may exist in which match play load negatively impacts hip flexibility. Verral et al. [26] suggested that a reduced total hip joint ROM is associated with later development of chronic groin injury and as such may be a risk factor. Screening of flexibility for football players should be conducted before football activities, and flexibility training should be mainly prescribed to players with reduced ROM to lower the risk of developing a muscle injury.

This study contains limitations that require acknowledgment. First, the only time-point selected to measure passive hip rotation ROM in response to a single football training or match was immediately post-training or post-match. Future studies should evaluate these variables using several time-points (until the next training) after the football activities. Second, it would be interesting to know the internal load caused by the training or the match and relate it to the changes of rotation in hip ROM. Third, a small sample was used, but this is often something that cannot be overcome when studying semi-professional football players with restricted bilateral hip ROM_RE_.

## 5. Conclusions

A single football training session or match leads to significant changes in hip rotation ROMs in players with restricted bilateral hip ROM_RE_. However, these changes did not reach normal values. According to the study, these findings may suggest an unstable hip joint with a predisposition to musculoskeletal injuries and screening of hip flexibility for football players before football activities to prevent injuries.

## Figures and Tables

**Table 1:** Demographic features of the sample.

TitData	Total Sample (*n* = 28 players; 56 limbs)	Normal Hip (NH Group) (*n* = 14 players; 28 limbs)	Restricted Hip (RH Group) (*n* = 14 players; 28 limbs)	*p* Values
Mean Age (years)	25 (4.30)	26 (4.76)	24 (4.04)	0.36
Height (cm)	181.00 (0.03)	183.00 (0.03)	181.00 (0.04)	0.39
Weight (kg)	76.71 (5.90)	77.44 (5.92)	75.21 (5.94)	0.18
Body Mass Index	22.14 (4.94)	23.19 (1.41)	21.50 (6.14)	0.43
Football Years	19.42 (4.68)	20.11 (4.37)	19.00 (4.97)	0.58

Data are reported as mean (SD).

**Table 2:** Relative differences and qualitative outcomes in passive hip rotation measurements for both limbs after training.

Variable	Pre-Training	Post-Training	*p* Value	Effect Size (d)
**NH Group**				
ROM_IR_ (°)	37 ± 4.27	38 ± 5.02	0.59	0.2
ROM_ER_ (°)	37 ± 2.20	35 ± 3.48	0.23	0.7
ROM_TOT_ (°)	74 ± 4.79	74 ± 6.32	0.95	0.0
ROM_REL_ (°)	1 ± 4.81	2 ± 5.90	0.38	0.2
**RH Group**				
ROM_IR_ (°)	37 ± 2.64	39 ± 3.64	0.01	0.6
ROM_ER_ (°)	29 ± 2.44	34 ± 3.38	0.001	1.96
ROM_TOT_ (°)	67 ± 3.75	73 ± 4.89	<0.001	1.37
ROM_REL_ (°)	9 ± 4.31	6 ± 4.74	0.07	0.7

Data are reported as mean ± standard deviation (SD). ROM_IR_: range of motion of internal rotation; ROM_ER_: range of motion of external rotation; ROM_TOT_: total rotation; ROM_REL_: relative internal rotation. Level of significance *p* < 0.05.

**Table 3:** Relative differences and qualitative outcomes in passive hip rotation measurements for both limbs after a match.

Variable	Pre-Match	Post-Match	*p* Value	Effect Size (d)	
**NH Group**					
ROM_IR_ (°)	40 ± 3.19	41 ± 5.01	0.66	0.2	
ROM_ER_ (°)	36 ± 1.87	35 ± 2.84	0.32	0.4	
ROM_TOT_ (°)	76 ± 3.97	76 ± 6.43	0.89	0.0	
ROM_REL_ (°)	4 ± 3.41	5 ± 5.01	0.23	0.2	
**RH Group**					
ROM_IR_ (°)	38 ± 3.84	37 ± 4.22	0.90	0.3	
ROM_ER_ (º)	29 ± 3.32	32 ± 2.79	*0.003*	1.0	
ROM_TOT_ (°)	68 ± 5.31	69 ± 5.81	*0.03*	0.2	
ROM_REL_ (°)	8 ± 4.84	5 ± 4.17	*0.05*	0.7	

Data are reported as mean ± standard deviation (SD). ROM_IR_: range of motion of internal rotation; ROM_ER_: range of motion of external rotation; ROM_TOT_: total rotation; ROM_REL_: relative internal rotation. Level of significance *p* < 0.05.

**Table 4:** Results from the between-group analysis in hip rotation ROMs.

Between Group Mean Difference of Changes in ROMs		
NH Group vs. RH Group	Training	Match
ROM_IR_ (°)	*p* = 0.27; *d* = 0.2	*p* = 0.53; *d* = 0.9
ROM_ER_ (°)	*p* = 0.001; *d* = 0.3	*p* = 0.01*; d* = 1.1
ROM_TOT_ (°)	*p* = 0.006*; d* = 0.2	*p* = 0.35; *d* = 1.1
ROM_REL_ (°)	*p*= 0.36*; d* = 0.7	*p* = 0.03*; d* = 0.2

Standard changes in mean (90 %CL). ROM_IR_: range of motion of internal rotation; ROM_ER_: range of motion of external rotation; ROM_TOT_: total rotation; ROM_REL_: relative internal rotation. Level of significance *p* < 0.05.
